# Sustainable Jam with Apple Pomace: Gelling, Rheology, and Composition Analysis

**DOI:** 10.3390/gels10090580

**Published:** 2024-09-08

**Authors:** Ândria Viegas, Maria João Alegria, Anabela Raymundo

**Affiliations:** 1 LEAF—Linking Landscape, Environment, Agriculture and Food Research Center, Associate Laboratory TERRA, Instituto Superior de Agronomia, Universidade de Lisboa, 1349-017 Lisbon, Portugalanabraymundo@isa.ulisboa.pt (A.R.); 2 SUMOL + COMPAL Marcas S.A., 2780-179 Carnaxide, Portugal

**Keywords:** response surface methodoloy, hydrocolloids, apple pomace, sustainability

## Abstract

Fruit juice processing can generate significant waste, but efficiently repurposing some of its byproducts not only reduces environmental impact but also adds value, thereby enhancing sustainability in the food industry. This work assesses the use of hydrocolloids in jam preparation and the influence of time and temperature on gelation in the presence of apple pomace. The effects of different processing conditions were analyzed using response surface methodology. Viscosity, elastic modulus (G′), viscous modulus (G″), and firmness were measured. Results indicated that both time and temperature significantly improved rheological and textural properties. The optimal conditions (35.6 min and 84.2 °C) yielded a viscosity of 3.66 × 10⁴ ± 4.49 × 10^2^ Pa·s and a G′ at 1 Hz of 2596 ± 128 Pa. The final product exhibited the desirable texture, was free of added sugars, had low lipid content, and retained its bioactive compounds. Applying apple pomace in the formulation allows a more efficient hydrocolloid system, promotes a circular economy, and combats food waste.

## 1. Introduction

Concerns about ecosystem sustainability continue to grow, leading companies to redefine their behaviors and processes to reduce food waste. The huge volume of fruit wastes is now a concern for waste management challenges, especially when taking the number of malnourished people and natural source depletion into consideration [1].

According to the United Nations Environment Program [2], in 2022, 1.05 billion tonnes of food were wasted. The processing of fruits and vegetables typically generates a large quantity of residual byproducts, constituting approximately 25% to 30% [3]. Residual byproducts may include pulp, pomace, peels, stems, and seeds. Many of these can pose environmental problems but can also be important sources of nutrients and bioactive compounds, including minerals, vitamins, dietary fibers, phenolic compounds, prebiotic oligosaccharides, and carotenoids [4,5]. The use of fruit byproducts presents opportunities for the development of functional food products. In addition, this use helps in the recycling of waste, promoting sustainable development and a greener environment [6]. Apples (*Malus* spp.) are one of the most popular fruits worldwide. They offer health benefits to consumers, including aiding in the prevention of chronic heart and vascular diseases, respiratory and pulmonary diseases, diabetes, obesity, or cancer, among others. Pomace, one of the edible byproducts in the processing of clarified apple juice, has been highlighted as a source of fiber and antioxidant compounds. Global production of apple pomace is estimated at an average of 4 million/year and is expected to continue to increase. Currently, the recovery rate of this byproduct is low. It is usually disposed of directly in the soil in a landfill, which can promote soil and water pollution because pomace is rich in water, sugars, and organic acids, making it an optimal environment for initiating microbial fermentation [7].

Most apple compounds remain in the pomace, including insoluble carbohydrates (cellulose, hemicellulose, pectin, and lignin), simple sugars, as well as small amounts of acids, minerals, proteins, and vitamins [8]. Natural pectin in fruits and vegetables forms a component of the food and acts as soluble dietary fiber. It can be extracted with at least 80% esterification [9], mostly finds use as a gelling agent in jams and jellies, and is known to effectively stabilize fruit juices and acidified milk drinks, high protein fruit drinks, and antioxidant-fortified foods [10]. Commercial pectin products are classified as high (>50%) or low (<50%) methoxyl pectin based on their degree of methyl esterification (DE), with the European regulation requiring at least 65% α-D-galacturonic acid [11]. Hydrocolloids are utilized in food due to their characteristics, such as thickening, gelling, controlling syneresis, stabilizing emulsions or suspensions, acting as a coating, and binding water.

**Figure 1:**
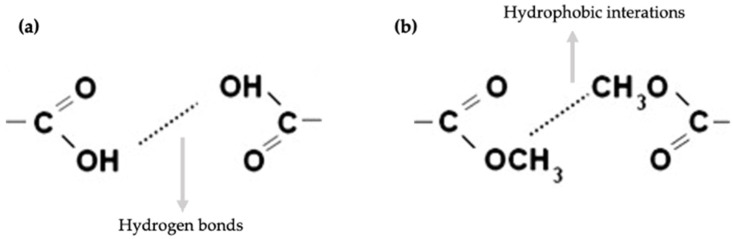
Intermolecular (**a**) hydrogen bonds and (**b**) hydrophobic interaction involved in high methoxyl pectin gelation. Adapted from Picot-Allain et al. [6].

The gelation process in jams naturally occurs during the cooling phase following high-temperature heating, such as in cooking. Initially, temperatures exceeding 50 °C are necessary to dissolve pectin in water. During heating, hydrophobic interactions arise between the non-polar methoxyl/ester groups in the pectin polymer chains. These interactions, represented in Figure 1, stem from the unfavorable relationship between water molecules and methoxyl groups, which disrupts the water structure and decreases its entropy. In response, the methoxyl groups aggregate, reducing their exposure to water. As the solution cools, gelation is triggered. The hydrophobic interactions (Figure 1b) weaken and disintegrate, permitting the formation of junction zones. These zones are stabilized by hydrogen bonds (Figure 1a) between the hydrophilic carboxyl groups (-COOH) on the pectin chains and the hydroxyl groups (-OH) of adjacent molecules. This network of interconnected chains forms a filamentous structure, resulting in the characteristic gel texture of jams [12].

Hydrocolloids are water-soluble polymers that have the ability to thicken, gel, or stabilize aqueous systems. The thickening properties of hydrocolloids vary depending on the type of hydrocolloid, its concentration, the food system in which it is used, as well as the pH and temperature of the process [11,13]. In particular, xanthan gum is highly versatile and demonstrates stability across a pH range of 2 to 12, along with a high water-binding capacity and solubility in both cold and hot water. Its viscosity behavior is fully reversible between 10 °C and 80 °C. These attributes contribute to heat stability, uniform viscosity, and excellent thermal stability for fruit fillings, particularly in baked goods, when exposed to high temperatures, and can serve as an excipient when used alone or in combination with other polymers [9,10]. The rheological behavior of hydrocolloids can be significantly influenced by factors such as shear rate, temperature, pressure, and duration of shearing. In Newtonian fluids, viscosity remains constant at a given temperature and pressure regardless of the shear rate. Conversely, in most non-Newtonian fluids, viscosity decreases as the shear rate increases, exhibiting shear-thinning characteristics. It is understood that this type of behavior reflects an irreversible structural breakdown, with viscosity declining due to molecular alignment occurring within the substance [14].

Jams are used as preserves for fruits with a high sugar content. These are prepared by boiling the fruit pulp with sugar, pectin, acid, and other ingredients (preservatives, colorings, and flavorings) until a reasonably thick gel consistency is obtained. In addition to the fruit itself, sugar is the main ingredient in jams. However, growing concerns about health, together with a higher incidence of diseases related to obesity and diabetes, have promoted an increase in demand for products with reduced or no added sugar, leading manufacturers to develop healthier alternatives to sugar [15]. The rheological properties of jam are mainly affected by the amount and type of sugar added, proportion and type of gelling agent used, fruit pulp content, and process temperature. These properties are useful in determining ingredient functionality in product development, quality control, and correlation of food texture with sensory attributes [16].

The objectives of this work are divided into three parts: First, investigate how hydrocolloids, temperature, and time affect the gelation process in jam. The second objective is to explore the use of apple pomace, an industrial byproduct of fruit processing, to promote a circular economy, and, finally, to develop a lower-calorie, sugar-free jam alternative that is packaged in tubes for easier use and improved preservation. These goals highlight both the scientific and practical significance of the work, aiming to drive innovation in food processing while advancing sustainability and promoting consumer health.

## 2. Results and Discussion

### 2.1. Jam Production and Control Selection

To achieve a formulation similar to a commercial jam (target), gels were produced with hydrocolloid concentrations between 0.25% and 0.75% (*w*/*w*), using various proportions of pectin and xanthan gum. The results are shown in Table 1.

Based on the results, it is observed that the firmness of the simple systems containing pectin and xanthan gum does not differ significantly from the control (*p* > 0.05). The only gel that showed significant differences compared to the target was the mixed system 0.5%XG + 0.25%P (0.053 N). When evaluating the impact of increasing the concentration of hydrocolloids in simple systems, it was found that for pectin, there were no significant increases in firmness with the increase in concentration. In the study carried out by Mohammadi-Moghaddam et al. [17], an opposite behavior to the present study was observed. The authors prepared a black plum peel jam and varied the pectin concentration between 0% and 0.4%, obtaining firmness values between 0.65 and 2.21 N. However, in the xanthan gum systems, there was a significant increase in firmness between the gel with 0.25% and the gel with 0.75% XG. The presence of xanthan gum can help stabilize the pectin network, forming a stronger matrix, more resistant to deformation. The interaction between the pectin and xanthan gum chains can occur through hydrogen bond interactions, increasing the cohesion of the gel [18]. The study conducted by Garrido et al. [19] showed that the firmness of apple pectin and xanthan gum hydrocolloids increases substantially with the increase in XG dosage.

Regarding the linear viscoelastic behavior, all samples presented similar behavior, with G′ greater than G″ and dependent on the oscillation frequency, typical of a weak gel-like structure [20]. Values of G′ at 1 Hz were used to compare the samples. It was found that, compared to the target sample (G′ at 1 Hz = 419 ± 6.2 Pa), all formulations present higher G′_1Hz_ values. It was expected that the commercial jam would have higher values since it contains sugar in its composition (the second ingredient in the list), while the formulations under study did not have any added sugars or sweeteners. It is known that sugar reduces water activity, promoting hydrophobic interactions, and, at the same time, stabilizes the junction zones by promoting interaction between the methyl ester groups of pectin. The concentration of sugar affects the strength of this gel network, which in turn influences the viscosity and texture of the final product. Generally, higher sugar concentrations lead to thicker and firmer jams. Previous studies on cherry and sapodilla jams have demonstrated that viscosity increases with higher sucrose concentrations [21].

However, considering the composition of the gels under study, where the primary component is apple pomace, it is important to note that this byproduct contains a significant amount of dietary fiber. Rocha Parra et al. [22] reported a dietary fiber content of 41.04 g/100 g dry weight (DW), while Aydodu et al. [23] found a total fiber content of 75.02 g/100 g DW.

The main components of dietary fiber are cellulose, lignin, hemicellulose, pectin, gums, and starch. The differences in these values could be related to the use of different cultivars and varieties, the maturation stage of the harvest, and processing steps [24]. Dietary fibers have high water-holding capacities, which contribute to the thickening of the gel. The absorbed water is trapped within the fiber matrix, increasing the viscosity and preventing syneresis (water separation) [25]. Figueroa and Genovese [26] evaluated the effect of the addition of dietary fiber from different sources (apple, bamboo, psyllium, and wheat) and pectin mass fraction (0.4 and 0.5 g/100 g) on the physicochemical properties of pectin gels for the development of a product similar to a fruit jam. When they fixed the concentration of pectin and increased the amount of fiber, they observed an increase in G′ in comparison with the control gel. Two possible explanations were proposed, namely: the soluble fraction of the fiber had a favorable effect on the gelation mechanism, contributing to the aqueous phase composition, and the insoluble fiber acted as a filler of the gel, reinforcing its structure.

Regarding the concentrations used, no significant increase in G′ was observed with the increase in XG. The results obtained are not in agreement with the study performed by Brunchi et al. [20] about the effects of concentration and temperature of a system based on xanthan gum since this author verified an increase in G′ with the increase in xanthan gum concentration from 0.4% to 2%, which may be related to the use of a low hydrocolloid concentration.

For the pectin concentration, the increase in G′ was only significant (*p* < 0.05) between the gels with 0.25%P and 0.75%P. The gelation mechanism of pectin is produced by non-covalent bonds of adjacent pectin chains, leading to an interconnected three-dimensional network [19]. These bonds are produced at junction zones, which are stabilized by hydrogen bonds and hydrophobic interactions between the methyl ester groups of the pectin chains [19]. A similar behavior was observed in the study carried out by Dervisi et al. [27] with the development of strawberry jam with six concentrations of pectin (0.1%, 0.5%, 2.5%, 5%, 7.5%, 10%) where they observed that as the pectin concentration increased, the value of the storage modulus also increased.

Figueroa and Genovese [26] also investigated the impact of increasing pectin concentration and found that the G′ of the gels with the same type of fiber increased with higher pectin content because the gel network is denser and has more junction zones, resulting in a stronger pectin network and a firmer gel structure. Another factor that may influence the difference between the gels under study and the commercial ones is pH. The pH value is critical to successful gel formation with pectin, and this is a key factor for the preservation and shelf-life of the product.

Apple pomace naturally has an acidic pH, typically ranging from 3.8 to 4.1. In the formulated products, pH ranges from 3.6 to 3.8. Pectin, being an anionic polysaccharide, undergoes protonation of carboxylic groups at lower pH levels, consequently diminishing electrostatic repulsions within and between pectin chains. Additionally, under low pH conditions, non-dissociated carboxylic groups engage in the formation of both inter and intramolecular hydrogen bonds with secondary alcohol groups [28].

In terms of flow behavior, all of the samples showed a shear thinning behavior, with a zero shear rate limiting viscosity, ƞ_0_ The results of the studied gels showed lower values of ƞ_0_, when compared to the target (*p* < 0.05). The high fiber content also influences the viscosity of the gels. Kirbas et al. [24] evaluated the effects of various powdered pomaces on the properties of a gluten-free cake. They found that viscosity increased with the increasing pomace content. Dietary fibers have a high water-holding capacity. When the water-holding capacity of ingredients used in cake batters increases, the free water content facilitating the movement of particles decreases, and the apparent viscosity increases.

No significant differences in this parameter were observed between the simple systems nor with the increase in pectin and xanthan gum concentration. The mixed systems (P + XG) showed lower values of $\eta_{0}$. Some studies report different behaviors from those obtained here; however, the hydrocolloid concentrations used in those studies are higher than those in the present study. For example, in the study carried out by Yoon et al. [29], in the preparation of naked barley flour with several types of gums, it was found that with an increase in XG concentration between 0.3% and 0.6%, the viscosity increased from 0.43 to 0.54 Pa·s. The mango filling made by Razak et al. [13] also confirms the previous trend. The study carried out by Prakash et al. [30] of jellies with the incorporation of pectin showed higher viscosity values when compared to jellies with gum, namely guar gum.

Given the results obtained, the formulation chosen to advance in jam gelation optimization studies was the 0.5%P + 0.25%XG formulation, as it presented firmness without significant differences (*p* > 0.05) when compared to the formulation target and a lower G′ value, which is closer to the target.

### 2.2. Jam Production and Optimization

A rotatable design with 12 experimental runs was used in the Response Surface Methodology. The independent variables included x_1_—time and x_2_—temperature, as represented in Table 2. Given that polymer gelation is considered an intermediate state between solubility and precipitation, the gelation process should be guided by thermodynamic principles [31]. Managing time and temperature is also crucial for preserving sensory and nutritional quality. It can help retain the natural vitamins and antioxidants in the fruit, which are often sensitive to prolonged heat exposure. Additionally, shorter cooking times and lower temperatures reduce energy consumption, making the process more environmentally friendly and cost-effective [32,33].

The respective responses (dependent variables), zero-shear rate-limiting viscosity (ƞ_0_), G′ at 1 Hz, and firmness, and the results obtained are presented in Table 3. These parameters are important because they provide insight into the behavior of the gels, their structure, and texture, which will later have an impact on the packaging, stability, and acceptability of the product [34].

The experimental and predicted values are presented in Table 3, where discrepancies between them have been observed. RSM is a statistically structured experimental design that varies multiple factors simultaneously to assess their impact on a response variable. This methodology typically uses polynomial equations to model the relationship between independent variables and the response. However, these relationships can be more complex, which may lead to inaccuracies in the quadratic model’s predictions. Insufficient replication or variability at the central points can further hinder the model’s ability to capture data variability accurately, resulting in discrepancies between the predicted and observed values [35]. Additionally, the mathematical equations used in RSM are valid only within the defined experimental range for independent variables, such as time and temperature, and cannot be extrapolated beyond this range. A similar methodology was employed by Benali et al. [36] in producing jelly from date syrup. They investigated independent variables, including cooking temperature (50–190 °C), cooking time (5–25 min), °Brix (67.5–77.5°), and cooling temperature (5–25 min). Their study found that cooking temperature and time had the most significant impact on the texture of the final product, with a cooking temperature of 155 °C for 10 min, resulting in a jelly with desirable textural properties.

**Figure 2:**
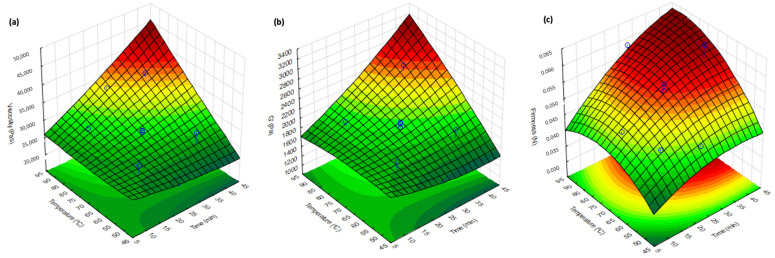
Response surface for the: (**a**) zero-shear rate-limiting viscosity (ƞ_0_); (**b**) G′ at 1 Hz; and (**c**) firmness of apple pomace jams prepared under different processing conditions (time and temperature).

The 3D response surface (Figure 2) shows that for the zero shear-rate limiting viscosity—ƞ_0_ (Pa·s), G′ at 1 Hz (Pa), and Firmness (N), there is a linear effect of time ($x_{1}$) and temperature ($x_{2}$) (*p* < 0.05).

The statistical analysis of the results is represented in Table 4. The adequacy of the model was assessed using the coefficient of determination (R^2^) and *p*-values for both the model and lack-of-fit tests. The lack-of-fit test compares the variation around the model to the inherent variation within replicated observations. A non-significant lack of fit indicates that the model is well-fitted and accurately represents the data [37]. The R^2^ value measures how much of the variability in the observed response values can be explained by the experimental variables and their interactions. The closer the R^2^ value is to 1, the better the model predicts the response. However, if pure error exists, it is impossible for R^2^ to reach 1. The results showed that for the three dependent variables, the adjusted R^2^ decreased compared to the R^2^. This decrease occurs when additional insignificant terms are added to the model, making it overly complex, or when there is insufficient data to support the model’s complexity [35].

The $\eta_{0}$ value increased linearly with time (*p* = 0.005) and temperature (*p* = 0.008). Quadratic effects and interaction effects between time and temperature were not significant (*p* > 0.05) and, as such, were not represented in the model Equation (1). The R^2^ value for the model was 0.8, the R^2^ adjusted was 0.63, and the *p*-value for the lack-of-fit test was 0.001 (*p* < 0.05). The significant lack of fit for viscosity indicates that the variance is a model-dependent measure of pure error, suggesting that the mathematical models may not adequately fit the experimental results [38].

(1) $$\eta_{0}=28,457.05+2093.52X_{1}+3332.58X_{2}$$

**Figure 3:**
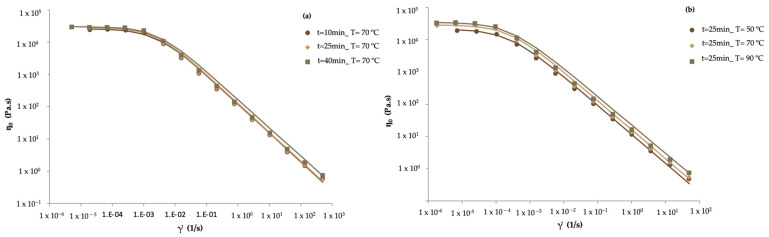
Evolution of the viscosity curves of apple pomace jams prepared with different: (**a**) stirring times and (**b**) processing temperatures.

The plot shows a gradient of colors from green to red, indicating increasing zero-shear rate-limiting viscosity values. Lower ƞ_0_ values are observed at lower temperatures and shorter times (bottom left corner of the plot). Higher viscosities are achieved at higher temperatures and longer times (top right corner of the plot). The highest viscosity is observed at the combination of the longest time and highest temperature studied (35.6 min and 84.2 °C), with a viscosity of 3.66 × 10^4^ Pa·s, and the lowest viscosity is observed at the combination of a moderate time and the lowest temperature studied (25 min and 50 °C), with a viscosity of 2.07 × 10^4^ Pa·s. The viscosity curves of jams are presented in Figure 3, with the respective Williamson’s model fit. All samples showed similar shear-thinning behavior, presenting a constant viscosity at low shear rates ($\eta_{0}$), followed by a continuous and gradual viscosity decrease with the shear rate increase. The obtained data for all tested jams was fitted using the Williamson model, showing regression coefficients (R^2^) between 0.986 and 0.999, thus suggesting a good fit for the chosen model. The linear viscoelastic behavior shown by these types of jams is similar to that found for pear pomace heating in the study carried out by Fernandes et al. [39].

At a constant temperature of 70 °C (Figure 3a), it was determined that at times 10, 25, and 40 min, the viscosity obtained was 2.49 × 10^4^, 2.86 × 10^4^, and 2.98 × 10^4^ Pa·s, respectively. In this way, it is possible to verify that with the increase in time, the difference between the values is not significant (*p* > 0.05); one possible justification is the factor of using a short time and temperature interval, with no significant increase between the star points and central points. At a constant time of 25 min (Figure 3b), the temperature was varied from 50, 70, and 90 °C and the viscosity obtained was 1.99 × 10^4^, 2.86 × 10^4^, and 3.39 × 10^4^, respectively. With increasing temperature, the increase in viscosity was significant (*p* < 0.05). These results are also in agreement with Falcão et al. [40], who studied the rheological behavior of grape jam with xanthan gum at different processing temperatures (45, 55, and 65 °C) where treatments 55 °C and 65 °C showed higher apparent viscosity in the range of shear rates studied when compared with the 45 °C treatment. The study performed by Khouryieh et al. [41] also showed that increasing the temperature from 25 °C to 80 °C promoted an increase in viscosity since heating xanthan to 80 °C would further disorder xanthan and increase its chain dimensions.

The gelation process in jams occurs naturally during the cooling phase from a high-temperature solution state (cooking process), where excess water is removed, thus allowing chemical interactions to occur. These interactions result in the formation of junction zones between closely aligned pectin chains. The junction zones are stabilized by hydrophobic interactions between the methoxyl groups and hydrogen bonds between protonated carboxyl and hydroxyl groups [34,42].

A frequency sweep test was used to determine the linear viscoelastic behavior of jam samples. The oscillation amplitude of stress was kept constant (3 Pa) within the linear viscoelastic region, previously accessed through the stress sweep tests. The frequency ranged from 0.01 to 100 Hz. In this test, the storage modulus (G′) and the loss modulus (G″) were obtained for the entire frequency range. The values of G′ at 1 Hz were used as the response in the RSM.

For G′ at 1 Hz, the same behavior was observed as in $\eta_{0}$. The statistical analysis of the results showed that G′ at 1 Hz increased linearly with time (*p* = 0.045) and temperature (*p* = 0.012). Quadratic effects and interaction effects between time and temperature were not significant (*p* > 0.05) and, as such, were not represented in model Equation (2). The R^2^ obtained was 0.78, and the *p*-value of the lack of fit obtained was 0.07. Considering a confidence level of 0.05, this value is not significant, which means the model is adequately adjusted.

(2) $$G\prime =1947.67+165.76X_{1}+232.96X_{2}$$

**Figure 4:**
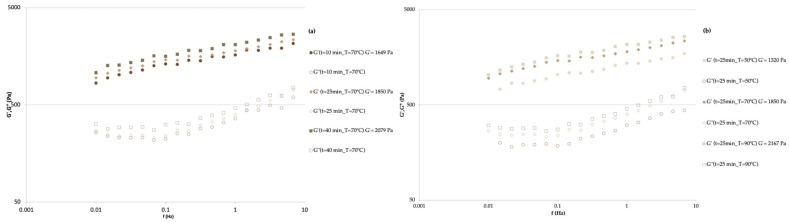
Evolution of the storage and loss moduli with frequency for apple pomace jam prepared with different: (**a**) stirring times and (**b**) temperatures.

The mechanical spectrum is represented in Figure 4, where the G′ was higher than G″ for all samples, and both parameters progressively increased throughout the studied frequency range. This behavior may be rheologically classified as a weak gel-like behavior, characteristic of fruit jams [20].

At a constant temperature of 70 °C (Figure 4a), it was determined that at times 10, 25, and 40 min, the G′ values obtained were 1649, 1850, and 2079 Pa, respectively. In this way, it was verified that with the increase in time, the G′ also increased (*p* < 0.05). At a constant time of 25 min (Figure 4b), the temperature varied from 50, 70, and 90 °C, and the G′ obtained was 1320, 1850, and 2167 Pa, respectively. With increasing temperature, the increase in G′ was significant (*p* < 0.05). Seshadri et al. [43] evaluated the rheological characteristics over time of an apple pectin solution, and found that at the initial time of the study, the gel had a highly viscous nature. As the gelation time progressed, the magnitude of the stress response of the pectin dispersion to the oscillatory sweeps increased, indicating that the material became more elastic in nature. The frequency dependence of G′ simultaneously became less pronounced. This represents a shift from a thick to an elastic behavior of the pectin dispersion. After 225 min, the G′ > G″ indicates that an elastic gel was formed. The results can be explained in terms of network formation between pectin molecules. Initially, no network has yet been formed, and each pectin molecule behaves very similar to a single dispersed molecule. As the pectin dispersion is cut, the molecules quickly relax due to their high molecular mobility. As time passes, pectin molecules begin to associate with each other due to the formation of hydrogen bonds and hydrophobic interactions.

Texture profile analysis (TPA), consisting of compressing a food sample twice, can be considered an imitation of the mastication operation. Firmness is defined as the force required to achieve a given deformation [21]. The statistical analysis of the results showed that firmness increased linearly with time (*p* = 0.022) and temperature (*p* = 0.003). Quadratic effects and interaction effects between time and temperature were not significant (*p* > 0.05) and, as such, were not represented in model Equation (3). The R^2^ obtained was 0.76, and the *p*-value of the lack of fit obtained was 0.03. Considering a confidence level of 0.01, this value is not significant, which means the model is adequately adjusted.

(3) $$Firmness=0.05451+0.00405X_{1}+0.00363X_{2}$$

**Figure 5:**
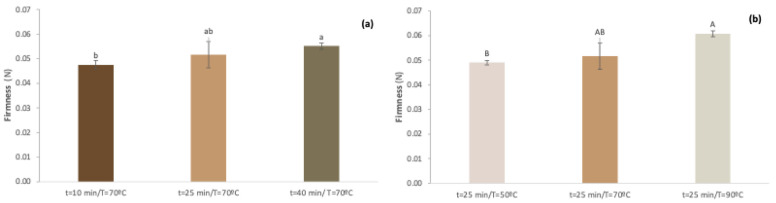
Firmness of apple pomace jam processing with different: (**a**) stirring times and (**b**) temperatures. Samples are presented as mean, with error bars indicating the standard deviations from the repetitions. In the same graph, different letters correspond to significant differences (*p* < 0.05).

At a constant temperature of 70 °C (Figure 5a), it was determined that at times 10, 25, and 40 min, the firmness obtained was 0.048, 0.052, and 0.055 N, respectively. In this way, it is possible to verify that with the increase in time, firmness also increased (*p* < 0.05). At a constant time of 25 min (Figure 5b), the temperature was varied from 50, 70, and 90 °C, and the firmness obtained was 0.049, 0.052, and 0.060 N, respectively. With increasing temperature from 50 °C to 90 °C, the increase in firmness was significant (*p* < 0.05). As shown in Figure 2c for firmness only, the linear effects of time and temperature were significant (*p* < 0.05). The study carried out by Zhang et al. [44] regarding sugar beet pectins showed that in a gel with pectin concentration fixed at 1.5% (*w*/*v*) and pH 3.5, the gel hardness increased significantly when treatment time increased from 30 to 120 min. In the study carried out by Benali et al. [36], it was shown that the higher the temperature or the longer the cooking time, the greater the hardness and stickiness of the date jelly.

The choice of process conditions largely depends on the desirable purposes of the product. If the objective is to minimize viscosity, the process must be carried out at lower temperatures and in shorter times. On the other hand, if a higher viscosity is desired, it would be beneficial to increase both temperature and time. The linear nature of the surface suggests that controlling the process can be relatively simple, as small adjustments in temperature and time will result in predictable changes in viscosity.

In the case of the present study, the objective is to package the gels in tubes. Tube products generally have a relatively high viscosity to prevent the contents from running or leaking. Proper viscosity also ensures that the product can be dispensed in a controlled manner when the tube is squeezed. Higher values of G′ contribute to better stability, ensuring that the product maintains its structure during storage and use. For the next stage of the study, the formulation was obtained under the processing conditions of t = 35.6 min and T = 84 °C. In these conditions, the highest viscosity value (3.66 × 10^4^ ± 4.49 × 10^2^ Pa·s) approaching the target formulation value was obtained. It also exhibits the highest G′ (2596 ± 128 Pa) and firmness value (0.062 ± 0.001).

### 2.3. Jam Characterization

The proximate composition (moisture, ash, protein, fat, and total sugar) of the apple pomace jam is present in Table 5, and a comparison with the results obtained by other authors with similar matrices is also presented.

In this sweet product, apple pomace, a byproduct generated during the processing for obtaining apple juice, was mainly used, and as such, for comparative purposes, a bibliography about apple pomace or apple pulp was used as reference.

The moisture content of this sweet product was determined as 15.29 ± 0.06 g/100 g dry weight (DW). The moisture content is crucial to consider when evaluating the stability and shelf life of apple pomace-based products. Since fresh pomace has a high moisture content, it can make the product susceptible to microbial growth and oxidation reactions. One way to prevent this is by drying the pomace. Dried pomace has been an option used in various products. Cantero et al. [48] first characterized apple pomace powder and assessed its feasibility for addition to gluten-free bread.

Furthermore, the ash content of apple pomace was measured as 2.73 ± 0.02 g/100 g DW, suggesting the presence of inorganic mineral components. Bhushan et al. [47] reported similar results ranging from 0.5 to 6.10 g/100 g; Wang et al. [45] and Jin et al. [46] presented lower values than the present study, 1.8% and 1.5%, respectively.

In terms of protein content, our analysis revealed that jam contains 4.00 ± 0.02 g/100 g DW of protein. This value is in accordance with the bibliography presented in Table 5. The presence of lipids was not very prominent, with a value of 0.29%, which, compared to the literature, is much lower.

As expected, the apple pomace jam contained a considerable amount of carbohydrates, 58.33 g glucose/100 g DW by the phenol sulfuric method, and by difference, the value obtained was 77.69 g/100 g DW; the results are not comparable, as they are represented in different units. In the bibliography found, the value obtained in this study is within the values presented by Bhushan et al. [47], corresponding to 48–62 g/100 g DW. According to this author, the predominant sugars in apple pomace were fructose (48.30%), followed by glucose (19.50–19.70%), arabinose and rhamnose (7.90%), and finally saccharose (3.80–5.80%). The fructose is a simple sugar that is sweeter than saccharose. This high sugar content highlights the sweet nature of apple pomace and suggests its suitability for use as a natural sweetener or flavor enhancer in various food products [49]. In the work carried out by Bhushan et al. [47], the value presented for the fiber was 4.70–51.10%. This compound is found in plant cell walls (non-starch polysaccharides) that resist digestion by human digestive enzymes. These fibers are recognized for their various protective effects on cardiovascular health, colorectal cancer, obesity, and diabetes.

The apple pomace composition is highly dependent on the manufacturing process, the apple cultivar, place of cultivation, state of maturation, and the year of harvest, which promotes the great variability of results [24].

Regarding the commercial jam that was considered as a target, its nutritional composition presents a lipid content of 0.4 g/100 g fresh weight (FW), carbohydrates of 40 g/100 g FW, of which sugars 39.3 g/100 g FW, and a protein value of 0.3 g/100 g FW. In order to make it possible to compare results, the values obtained in the present study, which are presented in Table 5, were converted to an FW, obtaining the following results: lipids: 0.04 g/100 g; carbohydrates (calculated by difference): 14.24 g/100 g FW, and protein: 0.6 g/100 g FW. It was found that the jam under study has a lower lipid content than the commercial jam and twice the protein content, in addition to not presenting any added sugars or sweeteners, presenting only the sugars naturally present in the fruit. Thus suggesting that it is a healthier alternative.

The mineral analysis presented in Table 6 showed high K (77.55 mg/100 g) and P (26.24 mg/100 g) content comparatively with the amounts of Na (8.64 mg/100 g), Ca (13.75 mg/100 g), and Mg (4.04 mg/100 g). According to Skinner et al. [50], both whole apples and apple pomace contain higher levels of potassium, sodium, calcium, and phosphorus, as observed in the present study. It has also been reported that pomace consistently exhibits higher values than the whole apple, probably due to the inclusion of the peel. Manzoor et al. [51] reported that the peel of apple cultivars had higher amounts of minerals compared to the pulp. Lomba-Viana et al. [52] also reported that pear pomace often had the highest values for several minerals when compared to puree and juice, as it is the fraction most concentrated in solids.

Potassium constitutes the predominant portion of the total mineral content of apple pomace (398.4–880.2 mg/100 g). Potassium plays a crucial role in reducing blood pressure, and a diet rich in potassium reduces the risk of cardiovascular or renal diseases. Sodium, phosphorus, and calcium are the next most abundant minerals in apple pomace, providing 183.5 mg/100 g, 64.9–70.4 mg/100 g, and 55.6–92.7 mg/100 g, respectively. Calcium and phosphorus are essential for bone health, and adequate intake can decrease the risk of osteoporosis [53]. Although apple pomace jam contains high amounts of minerals, these do not represent significant quantities for the product to have the nutritional claim “Source of” or “rich in”, in accordance with Regulation (EC) Nº 1924/2006 [54] and Regulation (UE) Nº 1169/2011 [55].

Table 7 shows the results for pH, soluble solids (°Brix), total phenolic compounds, and antioxidant activity for the apple pomace jams. The pH obtained was 3.67, slightly lower than that found in the literature. The °Brix obtained was 15.73, within the range obtained in the study carried out by Henriquez et al. [56].

The TPC was determined by the Folin–Ciocalteau method, and for antioxidant activity, the DPPH and FRAP methods were performed. The results obtained were lower when compared to the bibliography. For the TPC and DPPH method, the values obtained were 308.04 and 513.0, respectively. The results of the present study were compared with the study carried out by Lohani et al. [58]; although the solvent used in both works was methanol, it is worth noting that the method of extraction used was PEF, which can have a positive impact on the extraction and increase the value of phenolic and bioactive compounds. In the more detailed study carried out by Sudha et al. [57], it was possible to verify which compounds predominate in apple pomace, caffeic acid (60.28 mg/100 g) dominated, followed by epicatechin (24.63 mg/100 g) and quercetin (24.56 mg/100 g). Catechin (9.68 mg/100 g), gallic acid (5.46 mg/100 g), and chlorogenic acid (4.93 mg/100 g) were in appreciable quantities.

Regarding color, an L* of 62.86 ± 2.27, an a* of −5.43 ± 0.99, and b* value of 38.50 ± 1.63 were obtained for the final jam, and an L* of 44.1 ± 0.88, an a* of 13.16 ± 0.70, and b* of 33.98 ± 3.19, for the formulation target. The total color difference between these two formulations is 20.78, visible to the human eye [60].

## 3. Conclusions

Apple pomace is a byproduct of fruit juice processing with nutritional properties, including vitamins, minerals, carbohydrates, and fiber. It is a rich source of antioxidant activity and phenolic compounds and can be utilized to produce new food products. This study evaluated the effects of hydrocolloids, time, and temperature on the jam gelation process and their influence on the rheological characteristics of jam.

The experimental results indicate that increasing both time and temperature enhances the gelation process of the jam, leading to a significant increase in viscosity, firmness, and elastic modulus. The inclusion of hydrocolloids (xanthan gum and pectin) improved the ability to form viscous gels, resulting in a pleasant and consistent texture and higher viscosity in the final product. Additionally, the presence of fibers in apple pomace contributed to a better gel structure, demonstrating that a jam with good consistency can be achieved without the addition of sugars.

As a byproduct, apple pomace can play a significant role in the efficient use of biological resources, offering a cost-effective method for the nutritional and bioactive enrichment of foods.

Future work could explore the use of apple pomace in other food products, inspiring innovation in sustainable food processing and contributing to the development of healthier and eco-friendly products.

## 4. Materials and Methods

### 4.1. Preliminary Tests for Jam Production and Control Selection

For the production of jam, apple pomace and pulpy apple juice provided by the Portuguese company Sumol + Compal were used.

The preparation of the jam began with defrosting the apple pomace and pulpy juice overnight at 4 °C before mixing with other ingredients. The pulpy juice (30% *w*/*w*) and water (10% *w*/*w*) were heated until reaching the temperature of 70 °C. Then, the hydrocolloids were added, followed by the apple pomace (54–54.5% *w*/*w*). The addition of gelling agent pectin (P) and a thickener xanthan gum (XG) was also tested, with concentrations ranging from 0.25% to 0.75%, and in a mixed system (0.5% + 0.25%). Orange juice (5.15%) and cinnamon (0.10%) were then added. The ingredients were mixed in a food processor for 25 min at 70 °C, to achieve a final formulation with characteristics close to a commercial jam (target) with the following list of ingredients: Bravo de Esmolfe apple (55%), sugar, cinnamon (3.5%), gelling agent (pectin), and lemon juice.

### 4.2. Optimization of the Gelation Process

To optimize the gelation process, response surface methodology (RSM) was applied, considering as independent variables heating time (from 10 to 40 min) and the temperature (from 50 to 90 °C). The dependent variables considered were firmness (N), elastic modulus (G′) at 1 Hz (Pa), and zero-shear rate-limiting viscosity (Pa·s).

The design consists of factorial (−1, +1) and axial (−α, +α) points and four repetitions at the central point. In this trial, were used 12 assays (in triplicate). The generic mathematical expression is given by the Equation (4):

(4) $$Y=\beta_{o}+\beta_{1}\cdot X_{1}+\beta_{2}\cdot X_{2}+\beta_{12}\cdot X_{1}\cdot X_{2}+\beta_{11}\cdot X_{12}+\beta_{22}\cdot X_{22}$$

where Y is the response of the dependent variables, $\beta_{o}$ is the width of origin, $\beta_{1}$ and $\beta_{2}$ are the linear effects, $\beta_{11}$ and $\beta_{22}$ are the quadratic effects, and $\beta_{12}$ is the interaction effect between time and temperature.

### 4.3. Rheology Behavior

The rheological behavior (viscosity curves and mechanical spectra) of the formulations obtained in Section 4.1 and Section 4.2 was analyzed using a rotational rheometer the Haake Mars Modular Advanced Rheometer System (Thermo Scientific Haake MarsIII Controller, Waltham, MA, USA), connected with the Peltier cooling system and an Eheim professional air compression system. Samples were analyzed using a serrated parallel-plate geometry sensor with a 20 mm diameter and 2 mm gap at 20 °C. Amplitude oscillatory shear measurements were conducted over a frequency range of 0.01 to 100 Hz within the linear viscoelastic region that was previously determined by the stress sweep test. This allowed for the determination of the mechanical spectrum, such as the storage modulus (G′) and loss modulus (G″) as a function of frequency [39].

In order to obtain the flow curves, a shear rate of 10^−8^ to 500 s^−1^ was applied, using a serrated parallel plate system PP20 and 2 mm gap at 20 °C. This type of plate was used to prevent the sample from slipping. The obtained curves were adjusted to the Williamson model (Equation (5)).

(5) $$\eta =\frac{\eta_{0}}{1+{\left(k\dot{\gamma }\right)}^{m}}$$

where $\eta_{0}$ is the zero-shear Newtonian viscosity (Pa·s), k is the consistency coefficient, and m is the dimensionless shear thinning index.

### 4.4. Texture Profile

Texture Analyse was accessed according to the method described by Fernandes et al. [39] in the Stable Micro Systems texturometer—Texture Analyzer; TA–XT plus (Stable Micro Systems, Goldaming, UK), equipped with a 5 kg load cell and a cylindrical acrylic probe with 11 mm diameter. The test execution conditions included: pre-test speed 2.0 mm/s, post-test speed 2.0 mm/s, test speed 1 mm/s, and distance 8.0 mm at 20 °C The sample was placed in cylindrical glass containers with a diameter of 30 mm and a height of 40 mm, filled with a sample up to a height of 30 mm. The curves obtained in quintuplicate (force vs. distance) made it possible to determine the firmness.

### 4.5. Chemical Characterization

#### 4.5.1. Nutritional Composition

The dry matter was determined by weight loss (Equation (6)). Portions of 5 g samples were weighed into a crucible, previously weighed and tared, and the samples were dried in an oven at 105 °C (Binder GmbH, ED056, Tuttlingen, Germany), until constant weight. Finally, the crucibles were weighed after cooling in a desiccator.

(6) $$\mathrm{Dry}\;\mathrm{matter}\;\left(\%\right)=\frac{\mathrm{Dry}\;\mathrm{sample}\;\mathrm{weight}}{Fresh\;sample\;weight}\;\times \;100$$

To determine the ash content, the previously dried samples were placed in a muffle furnace (Snol) at 550 °C until they turned white [61].

The protein content of the samples was measured according to the Dumas method (Thermo Quest NA 2100 Nitrogen and Protein Analyzer, Interscience, Breda, The Netherlands). First, 70 mg of sample and 20 mg of VELP absorbent powder were weighed, in triplicate, into aluminum capsules. Subsequently, the sample was introduced into the equipment, where the sample was burned in the presence of oxygen. The result was expressed in grams of protein, using the conversion factor of 6.25 (indicated to Fruit Nectar).

Fat was assessed by the Soxhlet method. First, to the sample dried by freeze-drying for 72 h at −80 °C (ZIRBUS Technology GmbH, Bad Grund, Germany), 2.3 g of anhydrous sulfate (Sigma-Aldrich Chemical Company, St. Louis, MO, USA) was added in a thimble, and the extraction was performed using the Soxtec System HT 1043 extractor unit and 40 mL of petroleum ether as solvent. Extracted fat was finally dried (105 °C, 1 h), cooled, and weighted [52].

Total sugar content was measured by the phenol sulfuric method according to the method described by Pandey et al. [62], with some modifications. To a test tube, 0.5 mL of an aqueous solution of the extract (1 mg/mL) was added, followed by 0.5 mL of 5% phenol and 2.5 mL of sulfuric acid 96%. Next, the solutions were homogenized in a vortex and cooled at room temperature for 30 min. Finally, the absorbance of the samples was measured at 490 nm against distilled water. For the calibration curve, glucose was used in concentrations from 0 to 100 mg/mL. The obtained standard curve, represented in Equation (7), had a determination coefficient, R^2^, of 0.9835.

(7) y=0.0067x+0.0009

The mineral composition was determined by inductively coupled plasma optical emission spectrometry, following the method described by Lomba Viana et al. [52]. Firstly, 1 g of sample was digested using 12 mL nitric acid, 37% hydrochloric acid (HCl), and 4 mL of 65% nitric acid (HNO_3_). The digested mixture was added to demineralized water until reaching 50 mL of total volume. After sedimentation, the clarified was recovered and analyzed using the optical emission spectrometer.

#### 4.5.2. pH and Soluble Solids (°Brix)

pH determination was carried out by a pH electrode (50 10T, Hach, Rheineck, Switzerland) connected to the Basic 20 potentiometer (Crison, Alella, Spain). Readings were taken in triplicate with prior calibration using pH 4, 7, and 9 buffer solutions (Sigma-Aldrich).

The soluble solids content of the sample was evaluated by refractometry with a digital refractometer (Atago PAL-1, Tokyo, Japan).

#### 4.5.3. Color

For color measurement, a Chroma Meter CR-400 (Konica Minolta, Tokyo, Japan) was used, expressed in CIELAB. The colorimeter was calibrated with a white standard before use. The assays were performed at different sampling points until five similar points obtained in their three coordinates L*, a*, and b*. L* indicates luminosity, a* represents the color variation between green and red, and b* the color variation between blue and yellow. The color difference between the sample and target was determined by Equation (8).

(8) $$∆E=\sqrt[{2}]{{\left(L_{1}^{*}-L_{2}^{*}\right)}^{2}+{\left(a_{1}^{*}-a_{2}^{*}\right)}^{2}+{\left(b_{1}^{*}-b_{2}^{*}\right)}^{2}}$$

#### 4.5.4. Total Phenolic Compounds (TPC)

The gel samples were extracted (10% *w*/*v*) over 1 h at room temperature using a methanol/water solution (80:20 *v*/*v*). After 1 h, the samples were centrifuged at 10,000 rpm at 4 °C for 10 min.

The method adopted to determine TPC was that described by Matheus et al. [63] with some modifications to adapt the assay to 96-well microplates; 20 μL of extract (or gallic acid solution for the calibration curve) was placed on the microplate (NUNC96), followed by 100 μL of Folin–Ciocalteau reagent previously diluted in water (1:4 *v*/*v*). It was left to rest for 5 min, and then 80 μL of Na_2_CO_3_ (7%) was added to the solution. The reaction takes place for 2 h at room temperature and in the absence of light. After this period, absorbance was measured at 760 nm in a microplate reader, Clariostar plus (BMG Labtech, Ortenberg, Germany). Each sample was analyzed in triplicate. The calibration curve was carried out with gallic acid in water, with concentrations ranging from 0 mg/mL to 150 mg/mL. The obtained standard curve, represented in Equation (9), had a determination coefficient, R^2^, of 0.9992, and the results were expressed as gallic acid equivalents (GAE).

(9) y=350.86x+0.057

#### 4.5.5. Antioxidant Activity

For the analysis of antioxidant activity by the DPPH and FRAP method, the same extraction process was used in Section 4.5.4. Each method provides unique information about the different mechanisms of action of the antioxidants present, allowing for a more comprehensive and reliable understanding of the overall antioxidant efficacy. The DPPH assay measures the ability of an antioxidant to donate electrons or hydrogen atoms to neutralize stable DPPH free radicals, while FRAP assay evaluates the ability of an antioxidant to reduce ferric ions (Fe^3^⁺) to ferrous ions (Fe^2^⁺) [64].

For the DPPH method adapted from Matheus et al. [63], 20 μL of supernatant (or standard solution of Trolox, for the calibration curve) and 180 μL of DPPH solution (Aldrich Chemical Company, Milwaukee, WI, USA) were added and left to react for 30 min shielded from light. Finally, the absorbance was read at 517 nm in a microplate reader (Clariostar plus, BMG Labtech), using methanol as a blank. The calibration curve was prepared with Trolox (Aldrich Chemical Company, Milwaukee, WI, USA), with concentrations between 10 and 130 mg/mL. The obtained standard curve had a determination coefficient, R^2^, of 0.9928, represented in Equation (10). The radical scavenging activity (RSA) was calculated by Equation (11).

(10) y=40,149x−10.301

(11) $$RSA(\%)=\frac{{Abs}_{blank}-A{bs}_{sample}}{A{bs}_{blank}}\times 100$$

where ${Abs}_{blank}$ is the absorbance value of 20 μL of methanol with 180 μL of DPPH solution; ${Abs}_{sample}$ is the absorbance value of the sample under study.

For the FRAP method adapted from Khemiri et al. [65], it was necessary to prepare the FRAP reagent. It consisted of mixing 0.3 M acetate buffer at pH = 3.6 with 2,4,6-Tris(2-pyridyl)-s-triazine (TPTZ 10 mM) and 20 mM ferric chloride solution in a 10:1:1 ratio. In each well of the microplate, 25 μL of supernatant (or standard solution of Trolox, for the calibration curve) and 175 μL of FRAP solution (previously heated in a water bath at 37 °C for 15 min) were added. It was left to react for 30 min, shielded from light, and the absorbance was read at 595 nm in a microplate reader Clariostar plus. The calibration curve was prepared with Trolox (Aldrich), with concentrations between 10 and 130 mg/mL. The obtained standard curve had a determination coefficient, R^2^, of 0.9948, as represented in Equation (12). The results were expressed in TEAC.

(12) y=365.19x−0.3506

### 4.6. Statistical Analysis

STATISTICA v. 10 software was used to obtain the response surfaces. ANOVA analysis was performed to determine significant differences between the means, using the Tukey test to compare more than two samples at a significance level of *p* < 0.05. To adjust the obtained rheological data to the Williamson model, in viscosity determinations, Origin 2019b (OriginLab) software was used.

## Figures and Tables

**Table 1:** Rheological parameters of firmness (N), storage modulus at 1 Hz (G′, Pa), zero-shear Newtonian viscosity ($\eta_{0}$, Pa·s), consistency coefficient (k), and dimensionless shear thinning index (m). Values are presented as mean ± standard deviation. For each column, values with the same letter are not statistically different (*p* > 0.05).

Formulation	Firmness (N)	G′_1Hz_ (Pa)	ƞ_0_ (Pa·s)	m	k (Pa·s)	R^2^
0.25% P	0.044 ± 0.001 ^bc^	1190 ± 84.1 ^c^	3.49 × 10^4^ ± 2.42 × 10^3 cde^	1.02 ± 0.04 ^ab^	545.46 ± 17.77 ^bcde^	0.998 ± 0.004
0.5% P	0.047 ± 0.002 ^bc^	1419 ± 25 ^bc^	3.77 × 10^4^ ± 4.41 × 10^2 cd^	0.96 ± 0.07 ^ab^	735.71 ± 107 ^b^	0.999 ± 0.001
0.75% P	0.049 ± 0.001 ^abc^	1575 ± 133.5 ^ab^	4.24 × 10^4^ ± 3.62 × 10^3 c^	1.04 ± 0.04 ^ab^	664.33 ± 52.37 ^bcd^	0.999 ± 0.001
0.25% XG	0.041 ± 0.001 ^c^	1504 ± 133.4 ^ab^	3.13 × 10^4^ ± 3.15 × 10^3 de^	1.22 ± 0.06 ^a^	564.29 ± 47.84 ^bcde^	0.998 ± 0.002
0.5% XG	0.042 ± 0.001 ^bc^	1693 ± 9.2 ^a^	3.54 × 10^4^ ± 7.71 × 10^3 cde^	0.94 ± 0.09 ^ab^	700.75 ±59.85 ^bc^	0.999 ± 0.001
0.75% XG	0.049 ± 0.001 ^ab^	1703 ± 33.6 ^a^	3.50 × 10^4^ ± 1.50 × 10^3 cde^	0.94 ± 0.01 ^ab^	502.79 ± 46.43 ^cde^	0.999 ± 0.001
0.5% XG + 0.25% P	0.053 ± 0.002 ^a^	1376 ± 6.5 ^c^	2.84 × 10^4^ ± 1.83 × 10^3 b^	1.01 ± 0.07 ^ab^	475.70 ± 37.55 ^de^	0.998 ± 0.002
0.5% P + 0.25% XG	0.043 ± 0.001 ^bc^	984 ± 15.1 ^abc^	2.15 × 10^4^ ± 1.90 × 10^3 e^	1.13 ± 0.13 ^a^	435.30 ± 56.72 ^e^	0.995 ± 0.004
Target	0.044 ± 0.006 ^bc^	419 ± 6.2 ^d^	5.55 × 10^4^ ± 7.45 × 10^2 a^	0.82 ± 0.01 ^b^	1272.3 ± 156.78 ^a^	0.999 ± 0.003

**Table 2:** RSM matrix and respective responses for jams obtained under different processing conditions—time (x_1_) and temperature (x_2_).

	Coded Value		Decoded Value	
	$x_{1}$ (Time)	$x_{2}$ (Temperature)	Time (min)	Temperature (°C)
Factorial points	−1	−1	14.4	55.8
	1	−1	35.6	55.8
	−1	1	14.4	84.2
	1	1	35.6	84.2
Star point	−1.414	0	10	70
	1.414	0	40	70
	0	−1.414	25	50
	0	1.414	25	90
Central Points	0	0	25	70
	0	0	25	70
	0	0	25	70
	0	0	25	70

**Table 3:** Comparison between experimental results and those obtained by the equation model.

Formulation		ƞ_0_ (Pa·s)		G′_1Hz_ (Pa)		Firmness (N)	
Time	Temperature	Experimental	Predicted	Experimental	Predicted	Experimental	Predicted
14.4	55.8	2.7 × 10^4^ ± 5.7 × 10^2^	2.3 × 10^4^	1880 ± 104	1697	0.046 ± 0.002	0.047
35.6	55.8	2.9 × 10^4^ ± 1.4 × 10^3^	2.7 × 10^4^	1959 ± 63	1748	0.049 ± 0.002	0.055
14.4	84.2	2.8 × 10^4^ ± 5.6 × 10^2^	2.8 × 10^4^	1957 ± 71	1882	0.047 ± 0.001	0.054
35.6	84.2	3.6 × 10^4^ ± 4.5 × 10^2^	3.4 × 10^4^	2596 ± 128	2494	0.062 ± 0.001	0.062
10	70	2.4 × 10^4^ ± 1.4 × 10^2^	2.6 × 10^4^	1649 ± 63	1771	0.048 ± 0.001	0.049
40	70	2.9 × 10^4^ ± 4.3 × 10^2^	3.1 × 10^4^	2080 ± 24	2240	0.055 ± 0.002	0.060
25	50	2.1 × 10^4^ ± 7.6 × 10^2^	2.4 × 10^4^	1357 ± 63	1577	0.045 ± 0.002	0.049
25	90	3.4 × 10^4^ ± 6.5 × 10^2^	3.3 × 10^4^	2167 ± 41	2233	0.061 ± 0.001	0.060
25	70	2.9 × 10^4^ ± 9.7 × 10^2^	2.9 × 10^4^	2024 ± 45	1948	0.054 ± 0.001	0.055
25	70	2.8 × 10^4^ ± 1.6 × 10^3^	2.9 × 10^4^	1919 ± 32	1948	0.054 ± 0.002	0.055
25	70	2.9 × 10^4^ ± 3.6 × 10^3^	2.9 × 10^4^	2000 ± 132	1948	0.056 ± 0.001	0.055
25	70	2.8 × 10^4^ ± 1.3 × 10^3^	2.9 × 10^4^	1850 ± 13	1948	0.044 ± 0.001	0.055

**Table 4:** Coefficients of coded factors and ANOVA analysis for the responses in the optimization of apple pomace jam.

Factor	ƞ_0_ (Pa·s)	G′_1Hz_ (Pa)	Firmness (N)
Intercept	2.84 × 10^4^	1948.67	0.054
$x_{1}$	2.09 × 10^3^ *	165.76 *	0.004 *
$x_{2}$	3.33 × 10^3^ *	232.96 *	0.004 *
$x_{1}$ ^2^	2.08 × 10^2 ns^	29.29 ^ns^	−0.000 ^ns^
$x_{2}$ ^2^	1.84 × 10^2 ns^	−21.39 ^ns^	−0.002 ^ns^
$x_{1}x_{2}$	1.76 × 10^2 ns^	140.08 ^ns^	0.001 ^ns^
R^2^	0.80	0.78	0.76
R^2^ Adjusted	0.63	0.60	0.56
Lack-of-fit (*p*-value)	0.001 ^ns^	0.07 *	0.03 **

* significant at 0.05 level; ** significant at 0.01 level; ns: not significant.

**Table 5:** Proximate chemical composition of apple jam on a dry basis. The results are the mean of triplicates, shown as the mean ± standard deviation.

	Present Study	Wang et al. [45]	Jin et al. [46]	Bhushan et al. [47]
Moisture (g/100 g DW)	15.29 ± 0.06	4.4	5.8	3.90–10.80
Ash (g/100 g DW)	2.73 ± 0.02	1.8	1.5	0.5–6.10
Protein (g/100 g DW)	4.00 ± 0.02	3.8	4.7	2.94–5.67
Lipids (g/100 g DW)	0.29 ± 0.02	3.8	4.2	1.20–3.90
Carbohydrates (g/100 g DW) *	77.69 *	45.1	83.8	48.0–62.0
Total Sugar (g glucose/100 g DW)	58.33 ± 0.59			

* Calculated by difference: 100 − (moisture + ash + protein + lipids).

**Table 6:** Mineral profile of apple pomace jam and recommended values. The results are the mean of triplicates, shown as the mean ± standard deviation and are reported as mg of mineral per 100 g of fresh matter.

Minerals	Recommended Values in mg/100 g Source of/Rich in	Present Study (mg/100 g Fresh Matter)
Sodium	-	11.51 ± 0.16
Potassium	300/600	103.28 ± 1.39
Calcium	120/240	18.32 ± 0.30
Magnesium	57/114	5.38 ± 0.11
Phosphorus	105/210	34.94 ± 0.60
Sulfur	-	3.99 ± 0.01
Iron	2.1/4.2	0.47 ± 0.13
Copper	0.15/0.3	0.06 ± 0.005
Zinc	1.5/3	0.03 ± 0.002
Manganese	0.3/0.6	0.09 ± 0.001
Boron	-	0.28 ± 0.004

**Table 7:** Antioxidant activity (DPPH and FRAP methods), total phenolic compounds, pH, and ºBrix of apple pomace optimized jam. The results are the mean of triplicates, shown as the mean ± standard deviation.

	Present Study	Other Studies	References
pH *	3.67 ± 0.01	3.9	[57]
°Brix *	15.73 ± 0.06	13.3–15.8	[56]
TPC (mgGAE/100 g DW)	308.04 ± 0.01	385.4–402.7	[58]
DPPH (TEAC µmol Trolox/100 g DW)	513.0 ± 0.07	780.6–799.3	[58]
FRAP (TEAC µmol Trolox/100 g DW)	1110.0 ± 0.01	3336–8525	[59]

* Fresh pomace.

## Data Availability

The original contributions presented in the study are included in the article; further inquiries can be directed to the corresponding author.
